# Cognitive Dysfunction in Asian Patients with Depression (CogDAD): A Cross-Sectional Study

**DOI:** 10.2174/1745017901713010185

**Published:** 2017-10-31

**Authors:** Srisurapanont Manit, Mok Yee Ming, Yang Yen Kuang, Chan Herng-Nieng, Della Constantine D, Zainal, Nor Zuraida, Jambunathan Stephen, Amir Nurmiati, Kalita Pranabi

**Affiliations:** 1 Department of Psychiatry, Faculty of Medicine, Chiang Mai University, Chiang Mai, Thailand; 2 Institute of Mental Health, View, Buangkok Green Medical Park, Buangkok, Singapore; 3 Department of Psychiatry, National Cheng Kung University, Cheng Kung National University Hospital, Tainan, Taiwan,; 4 Department of Psychiatry, Singapore General Hospital, Academia, Singapore; 5 College of Medicine, University of the Philippines, Manila, Philippines; 6 University Malaya, Jalan Universiti, Kuala Lumpur, Wilayah Persekutuan, Malaysia; 7 Department of Psychiatry, Cipto Mangunkusumo Hospital, University of Indonesia, Jakarta Pusat, Indonesia; 8 Lundbeck Singapore Pte. Ltd., 101 Thomson Road, Singapore

**Keywords:** Asia, Depression, Cognition, Functioning, Perceived cognitive dysfunction, Symptom

## Abstract

**Background:**

Cognitive dysfunction is a predominant symptom of Major Depressive Disorder (MDD), contributing to functional impairment.

**Objective:**

The primary objective of this study was to assess and describe perceived cognitive dysfunction amongst Asian patients diagnosed with MDD. The secondary objective was to explore the associations between depression severity, perceived cognitive dysfunction and functional disability.

**Methods:**

This was a multi-country, multi-centre, cross-sectional study. Adults with a current episode of MDD were recruited from 9 university/general hospital clinics in Asia. During a single study visit, psychiatrists assessed depression severity (Clinical Global Impression-Severity, CGI-S); patients completed questionnaires assessing depression severity (Patient Health Questionnaire-9 items, PHQ-9), perceived cognitive dysfunction (Perceived Deficit Questionnaire-Depression, PDQ-D) and functional disability (Sheehan Disability Scale, SDS).

**Results:**

Patients (n=664), predominantly women (66.3%), were aged 46.5±12.5 years, lived in urban areas (81.3%) and were employed (84.6%). 51.5% of patients were having their first depressive episode; 86.7% were receiving treatment; 82.2% had a current episode duration >8 weeks. Patients had mild-to-moderate depression (CGI-S=3.3±1.0; PHQ-9=11.3±6.9). Patients reported perceived cognitive dysfunction (PDQ-D=22.6±16.2) and functional disability (SDS=11.3±7.9). PHQ-9, PDQ-D and SDS were moderately-to-highly correlated (PHQ-9 and SDS: r=0.72; PHQ-9 and PDQ-D: r=0.69; PDQ-D and SDS, r=0.63). ANCOVA showed that after controlling for patient-reported depression severity (PHQ-9), perceived cognitive dysfunction (PDQ-D) was significantly associated with functional disability (SDS) (*p*<0.001).

**Conclusions:**

Asian patients with MDD reported perceived cognitive dysfunction. There is a need for physicians to evaluate cognitive dysfunction in the clinical setting in order to reach treatment goals, including functional recovery beyond remission of mood symptoms.

## 1 INTRODUCTION

Cognitive dysfunction is increasingly recognised as one of the core features of Major Depressive Disorder (MDD) [1], which has an impact on patients’ social and occupational functioning, as well as quality of life [2]. Cognitive symptoms in MDD include impairments in reasoning, problem solving skills, memory, social cognitive performance, and motor speed or attention [3]. In the Diagnostic and Statistical Manual of Mental Disorders, 5^th^ edition, cognitive symptoms in MDD are described as “indecisiveness” or the “diminished ability to concentrate” [4].

Treatment goals in MDD have evolved from the clinical remission of mood symptoms, to the recovery of premorbid social and occupational functioning [5]. For patients, returning to usual levels of functioning at work, home, or school are important criteria for remission [6]. Functional recovery may however be impeded by symptoms that persist despite improvement in affective symptoms [7]. Cognitive dysfunction in particular has been identified as one such symptom [2]. Specific patterns of aberrant cognitive processing, including difficulties with disengaging from negative material and deficits in cognitive control when processing negative material, have been posited to hinder recovery from depressive episodes thus resulting in a sustained negative affect [8].

Studies in the West, albeit heterogeneous in study design and assessments, have reported the presence of cognitive dysfunction in MDD, and suggest a link between depression severity, cognitive dysfunction and functional disability [9-12]. Various neurocognitive tests are available and have been employed in these studies [1]. In the clinical setting, however, the assessment of cognitive dysfunction from the patient perspective becomes particularly important, as cognitive impairments appear to be present early in the course of illness [1] and may first be detected by patients [13]. Due to the progressive nature of cognitive deterioration in MDD [14], it may take years before cognitive dysfunction is diagnosed using objective neurocognitive tests [13]. Hence, assessments of subjective cognitive dysfunction may be used as an early screener of potential cognitive dysfunction, thus allowing clinicians to tailor their treatment strategies based on the patient’s clinical profile.

Previous studies have been conducted in Asian MDD populations that assessed cognitive dysfunction; these included the Study on Aspects of Asian Depression (SAAD) and the Epidemiological Research on Functioning Outcomes Related to Major depressive disorder in South Korea (PERFORM-K) study. The SAAD was conducted in 6 countries in Asia (China, Korea, Malaysia, Singapore, Taiwan and Thailand) and assessed subjective memory and cognitive deficits using only two items of the Symptom Checklist-90-Revised tool (items 9 and 55 about the respondent's “trouble remembering things” and “trouble concentrating” during the week prior to assessment, respectively) [13]. The PERFORM-K study used a more comprehensive tool that assessed planning/organization, in addition to memory and concentration; however, this study was confined to patients in South Korea [15]. There are thus a limited number of studies that have been conducted in Asian population that comprehensively explored, either objectively or subjectively, the cognitive aspect of MDD in the local context.

The primary objective of this study was to assess and describe perceived cognitive dysfunction amongst Asian patients diagnosed with MDD. The secondary objective was to explore the associations between depression severity, perceived cognitive dysfunction and functional disability.

## 2 METHODS

This was a multi-country, multi-centre, cross-sectional study investigating perceived cognitive dysfunction in Asian patients with depression. Patients were recruited from 9 inpatient and outpatient psychiatry clinics from university or general hospitals in 6 Asian countries (Indonesia, Malaysia, Philippines, Singapore, Taiwan and Thailand) between 14 October 2014 and 8 May 2015. The study was approved by the institutional review board or the ethics committee of each site. Study patients gave written informed consent prior to their participation.

### 2.1 Patients

Patients who were between 21 and 65 years old, and assessed by psychiatrists to be clinically diagnosed with an active episode of MDD during the study visit were included in the study. Patients were excluded if they: had a concurrent diagnosis or past medical history of schizophrenia or other psychotic disorder, bipolar disorder, dementia or other neurodegenerative disease, alcohol or substance use dependence, or other psychiatric disorder; were study personnel or immediate family members of study personnel, or subordinates (or immediate family member of a subordinate) to any study personnel; were previously enrolled in the present study; or were unlikely to comply with the protocol in the investigator’s opinion.

The assignment of the patients to a therapeutic strategy was not decided in advance by the study protocol and was clearly separated from the decision to include the patients in the study.

### 2.2 Study Assessments

Patients were assessed by psychiatrists and completed patient-reported outcome (PRO) questionnaires during a single study visit.

Depression severity was assessed by psychiatrists using the Clinical Global Impression - Severity of Illness scale (CGI-S), a standardised, generic assessment tool to rate the severity of an illness on a 7-point scale (a score of 1 indicates normal health and a score of 7 indicates extreme illness) [16].

The 9-item Patient Health Questionnaire (PHQ-9) [17] was used by patients to rate the severity of their depression. A total score is calculated, ranging from 0 (absence of depression) to 27 (severe depression). Depression severity categories are: “no depression” (total score ≤4), “mild depression” (5-9), “moderate depression” (10-14), “moderately severe depression” (15-19) and “severe depression” (20-27). The PHQ-9 also includes a question to evaluate how difficult MDD makes working, taking care of things at home, or getting along with other people for patients, which were rated as “not difficult at all” (0), “somewhat difficult” (1), “very difficult” (2) or “extremely difficult” (3).

Patients assessed their cognitive dysfunction using the Perceived Deficits Questionnaire for Depression (PDQ-D) [18-20]. The PDQ-D consists of 20-items with four domains: attention/concentration, retrospective memory, prospective memory and planning/organization. Each item assesses how often the patient experiences a cognitive symptom in the past 7 days using a scale of 0 to 4: “never” (0), “rarely – once or twice” (1), “sometimes – 3 to 5 times” (2), “often – about once a day” (3) and “very often – more than once a day” (4). Each domain consists of 5 items (maximum total PDQ-D score for each domain was 20), adding up to a total PDQ-D score of 80 for all of the 4 PDQ-D domains. No score threshold has been defined for severity of perceived cognitive dysfunction; as such, categories were defined as distribution-based quartiles with higher scores indicating worse perceived cognitive dysfunction.

Patients assessed their functional disability using the Sheehan Disability Scale (SDS). The SDS assesses functional disability over the previous 7 days in 3 domains: work/school, social life/leisure activities and family life/home duties [21]. Patients rate the severity of disability in each domain on a scale of 0 to 10; an SDS domain score ≥4 indicated moderate, marked or extreme functional disability. A total functional disability score was computed ranging from 0 (no functional disability) to 30 (severe functional disability).

Socio-demographic data and medical history, including MDD history and management, were also collected during the study visit. There was no specific safety assessment beyond the routine reporting of adverse events.

### 2.3 Statistical Analyses

The population for the analysis was comprised of all eligible patients who received the patient information document, gave their informed consent, met the selection criteria and completed at least one PRO questionnaire.

Patient demographics and study assessments data were summarised using descriptive techniques. Continuous variables were presented as number of observations, mean ± standard deviation (SD), unless otherwise stated; categorical and binary variables were presented as counts and percentages. Statistical tests were two-sided at a 5% significance level.

Severity categories of perceived cognitive dysfunction were described as distribution-based quartiles of PDQ-D scores. Descriptive analyses were performed comparing mean PDQ-D scores by domain (attention/concentration, retrospective memory, prospective memory and planning/organization). In addition, mean total PDQ-D scores were compared between patient subgroups defined by age (21-25 years, 26-30 years, 31-35 years, 36-40 years, 41-45 years, 46-50 years, 51-55 years, 56-60 years, 61-65 years) and number of previous depressive episodes (1, 2, 3 or >3 previous depressive episodes). In this study, mean total PDQ-D score ≥20 was defined as clinically relevant perceived cognitive dysfunction (patients reported ≥1 for each of the 20 PDQ-D items).

Depression severity (mean CGI-S and PHQ-9 scores) and functional disability (SDS mean total score) were compared between patient subgroups defined by PDQ-D quartiles. Correlations between physician-assessed depression severity (CGI-S), patient-reported depression severity (PHQ-9), perceived cognitive dysfunction (PDQ-D) and functional disability (SDS) were calculated using Pearson’s correlation coefficients. Categories for Pearson’s correlation coefficients previously used in literature [22] and also used in this study were: “very high” (0.9–1.0), “high” (0.7–0.9), “moderate” (0.5–0.7), “low” (0.3–0.5) or “negligible” (0–0.3).

To identify factors associated with functional disability, functional disability (total SDS score) was first compared between patient subgroups using one-way analysis of variance (ANOVA). Patient subgroups were defined by the following variables: country, gender, age, marital status, education, recurrent depressive episode, history of hospitalisation, history of sick leave in the past 12 weeks, depression severity (CGI-S, PHQ-9), at least 1 chronic medical condition, at least 1 functional syndrome, at least 1 anxiety disorder, at least 1 concomitant mental disorder, anti-depressant treatment (initiated, switched or maintained), severity of perceived cognitive dysfunction as assessed by total PDQ-D score and scores for each PDQ-D domain. Factors associated with functional disability (total SDS score) in the ANOVA (at the 0.05 threshold) were then evaluated using analyses of co-variance (ANCOVA).

A total of three ANCOVA models were built to explore the associations with functional disability. ANCOVA model 1 was built using factors significantly associated (*p*<0.05) with functional disability (total SDS score) in the ANOVA. ANCOVA model 2 was then built without both PDQ-D and CGI-S. Finally, ANCOVA model 3 was built with PDQ-D, but without CGI-S. PHQ-9 was an adjustment co-variate in all 3 ANCOVA models.

For all outcomes presented herein, missing data were not replaced in the analyses. Analyses were performed using the SAS^^®^^ statistical software (SAS Institute, Cary, NC, USA), Version 9.2.

## 3 RESULTS

**Fig. (1):**
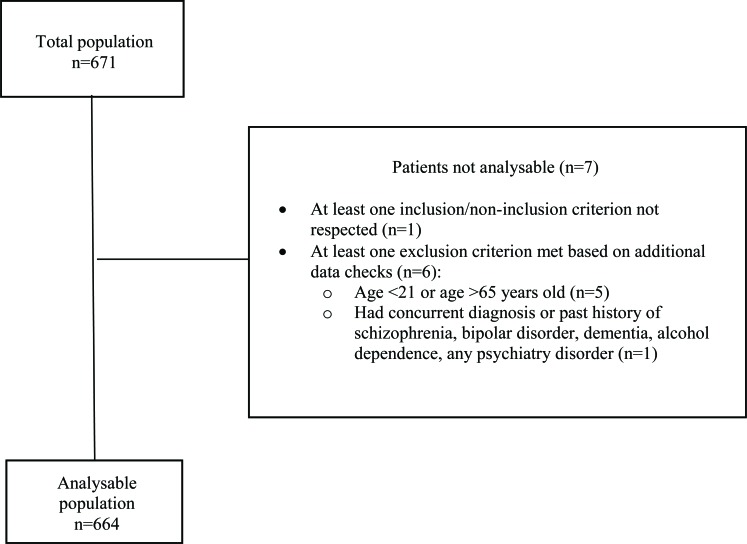
Patient flowchart.

A total of 671 patients were enrolled in the study, of whom 7 were excluded from the analysable population (Fig. **1**). The most common reason for exclusion was age (patient was above 65 years old or below 21 years old).

### 3.1 Patient Demographics

Patient demographics are presented in Table (**1**). The majority of patients (96.1%) were recruited from outpatient psychiatry clinics. Patients had a mean age of 46.5 ± 12.5 years, with 68.7% in the age group between 41 to 65 years. The majority of patients were women (66.3%), married or living as a couple (58.9%), living in urban areas (81.3%), and were working (including paid work, non-paid work such as charity work or volunteer work, or were self-employed), students or homemakers (84.6%). On average, patients had 12.8 ± 4.7 years of school, college or university education.

The clinical characteristics of the patients are reported in Table (**1**). The study visit was the first depressive episode in 51.5% of patients. In 82.2% of patients, the duration of the current depressive episode was more than 8 weeks since onset. The majority of patients (86.7%) were undergoing one or more pharmacological or non-pharmacological treatments for the current depressive episode prior the study visit; amongst these, 78.3% were undergoing one treatment. Treatments included: selective serotonin reuptake inhibitors (64.9%), serotonin-norepinephrine reuptake inhibitors (13.7%), tricyclic antidepressants (4.7%), and other antidepressants (agomelatine, bupropion, mianserin, mirtazapine, moclobemide, tianeptine, trazodone, other) (31.3%), as well as non-pharmacological treatments (psychotherapy, others) (4.9%). The reason for switch to another treatment during the study visit was primarily due to lack of efficacy of previous treatment (55.7% of switch patients).

More than half of the patients (55.9%) presented with clinically significant symptoms of anxiety and 21.2% of patients had comorbid anxiety disorders. Amongst patients who had a past history of depressive episodes (48.5% of total study population), 70.5% had the last depressive episode more than 12 months prior to the current depressive episode; 91.3% were treated in the last depressive episode, and 82% achieved clinical remission.

### 3.2 Depression Severity, Perceived Cognitive Dysfunction, Functional Disability

Psychiatrists assessed patients as having mild to moderate depression: 54.5% had “mild” depression and 31.2% had “moderate” depression (Table **2**). The mean total CGI-S score was 3.3 ± 1.0. Patients rated their own depression severity as “moderate”, with a mean total PHQ-9 score of 11.3 ± 6.9. For 78.9% of patients, MDD made working, taking care of things at home or getting along with people ‘somewhat’ to ‘extremely’ difficult. Patients in these categories had a corresponding mean total PDQ-D score ≥20, indicating clinically relevant perceived cognitive dysfunction.

Patients reported a mean total PDQ-D score of 22.6 ± 16.2 (Table **2**). There are no specific cut-off PDQ-D score ranges that define perceived cognitive dysfunction severity. In the study, observed PDQ-D score distributions by quartile were: 0–9 (1^st^ quartile), 10–19 (2^nd^ quartile), 20–31 (3^rd^ quartile) and 32–80 (4^th^ quartile). Half of the patients (51.7%) had a mean total PDQ-D score ≥20. Worse perceived cognitive dysfunction was reported in the domains of “Attention/Concentration” (6.21 ± 4.64) and “Planning/Organisation” (6.00 ± 4.93), than for memory (“Retrospective Memory”: 5.39 ± 4.41; “Prospective Memory”: 4.99 ± 3.88).

When mean total PDQ-D scores were compared between patient subgroups defined by age (21-25 years, 26-30 years, 31-35 years, 36-40 years, 41-45 years, 46-50 years, 51-55 years, 56-60 years, 61-65 years), worse perceived cognitive dysfunction was seen in patient subgroups between the ages of 21 and 40 (mean total PDQ-D score ranged from 26.2 ± 14.7 to 32.1 ± 12.9) than in patient subgroups between the ages of 41 and 60 (mean total PDQ-D score ranged from 15.1 ± 13.2 to 23.4 ± 16.2). Similarly, worse perceived cognitive dysfunction was reported in patients with 3 or >3 previous depressive episodes (mean total PDQ-D score was 30.3 ± 15.5 and 31.3 ± 15.7 respectively), compared to patients with 1 or 2 depression episodes (mean total PDQ-D score was 18.3 ± 14.6 and 19.4 ± 16.9, respectively).

Patients reported a mean total SDS score of 11.3 ± 7.9 (Table **2**). Patients also reported a mean of 1.33 ± 2.33 days lost and a mean of 2.39 ± 2.69 unproductive days in the previous 7 days. A mean SDS domain score ≥4 (indicating ‘moderate’, ‘marked’ or ‘extreme’ disruption) was reported by 51.9%, 49.7% and 47.3% of the study population in the subdomains of ‘work/school’, ‘social life/leisure activities’ and ‘family life/home responsibilities’, respectively. Patients in these functional disability categories had a corresponding mean total PDQ-D score ≥20, indicating clinically relevant perceived cognitive dysfunction.

### 3.3 Associations Between Perceived Cognitive Dysfunction, Depression Severity and Functional Disability

**Fig. (2):**
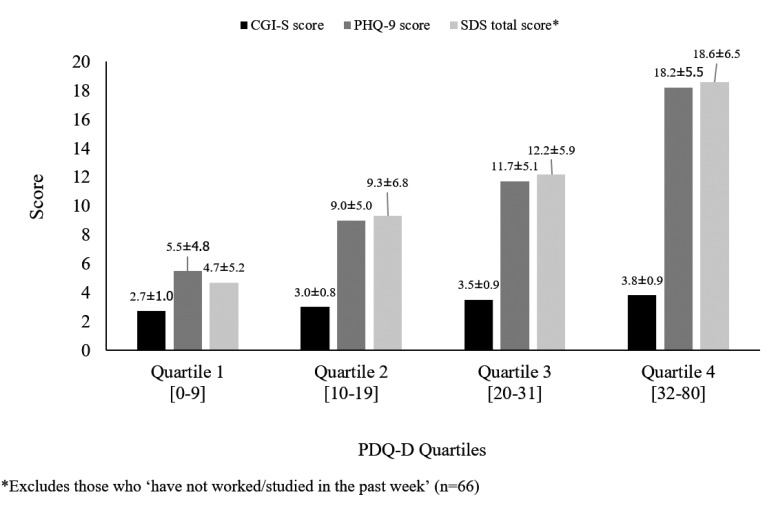
Mean CGI-S, PHQ-9 and total SDS scores by PDQ-D categories.

Patients with worse depression severity (higher mean CGI-S and PHQ-9 scores) had more severe perceived cognitive dysfunction, and patients with worse perceived cognitive dysfunction reported greater functional disability (Fig. **2**).

These results were confirmed by Pearson’s correlation analyses, which showed moderate-to-high correlations between depression severity, perceived cognitive dysfunction and functional disability (PHQ-9 and SDS: r=0.72; PHQ-9 and PDQ-D: r=0.69; PDQ-D and SDS, r=0.63).

Factors identified by ANOVA to be associated with functional disability (*p*<0.05 for association between factor and at least one SDS sub-score or total SDS score) included: country, gender, age, marital status, education, recurrent depressive episode, hospitalisation, history of sick leave in the past 12 weeks, depression severity (CGI-S, PHQ-9), at least 1 chronic medical condition, at least 1 functional syndrome, anti-depressant treatment (initiated, switched or maintained), and severity of perceived cognitive dysfunction as assessed by total PDQ-D score and scores for each PDQ-D domain (Table **3**).

ANCOVA was used to further understand the factors associated with functional disability (total SDS score). The first ANCOVA model showed that ‘PDQ-D’, ‘country’ and ‘recurrent depressive episode’ were independently associated with total SDS score (*p*=0.000, *p*=0.003, *p*=0.013, respectively) (Table **4**), with PHQ-9 as the adjustment covariate.

The second ANCOVA model assessed associations of other variables with total SDS score in the absence of PDQ-D and CGI-S, while keeping PHQ-9 as the adjustment covariate. The results showed that ‘country’ and ‘recurrent depressive episode’ remained associated with total SDS score (*p*=0.000 and *p*=0.01, respectively) (Table **4**).

In the final ANCOVA model, PDQ-D was included (CGI-S was excluded), with PHQ-9 kept as the adjustment covariate. After controlling for PHQ-9, PDQ-D was significantly associated with total SDS score (*p*=0.000) (Table **4**). Significant differences in mean total SDS score were also reported between countries (*p*=0.001), as well as between patients in their first episode of depression and patients in a recurrent depressive episode (*p*=0.021).

## 4 DISCUSSION

This is one of the few observational studies to assess and describe perceived cognitive dysfunction in patients with MDD across multiple countries in Asia. Results of this study add to the existing knowledge base on the presence of cognitive dysfunction in patients with MDD and its associations with depression severity and functional disability. Furthermore, while previous Asian studies have characterised the clinical features of patients with MDD in Asia, patients with MDD in these studies had worse depression severity compared to patients in the present study [13, 15]. Results of our study, which covered a wide geography within Asia, thus provide a different perspective on depression severity, perceived cognitive dysfunction and functional disability in a population with lower depression severity at a later stage of their current episode with higher treatment rates, and allows an exploration of the associations between these factors with perceived cognitive functioning and functional disability.

Patients with MDD in our study had mild-to-moderate depression severity as assessed by psychiatrists using the CGI-S. Patients had less severe depression than patients in previous Asian studies, such as the SAAD study conducted in 6 Asian countries (n=547), in which 89.8% of patients had moderate-to-severe depression as assessed by the Montgomery–Åsberg Depression Rating Scale (MADRS) (mean MADRS score: 29.1 ± 8.1) [23]. Patients with MDD from the PERFORM-K study in South Korea (n=312) also had moderate-to-severe depression (mean total CGI-S score: 4.3 ± 0.9; mean total PHQ-9 score: 16.0 ± 6.5) [15]. The higher CGI-S and PHQ-9 scores in PERFORM-K compared to the present study may be related to the inclusion of patients with MDD who either required treatment or switched treatment during the study visit, which suggested that patients with MDD were having active symptoms or were refractory to previous treatment. In PERFORM-K, 22.8% of patients were receiving treatment at the time of inclusion in the study and 34.3% of patients had less than 8 weeks’ duration of their current episode (compared to 86.7% and 17.8% in the present study, respectively). Furthermore, the majority (96.1%) of patients in our study were seen in the outpatient setting. The results of the present study may therefore be reflective of a patient population at a later stage of their current episode, with lower depression severity and higher treatment rates, than in the SAAD and PERFORM-K studies.

Our study confirms findings from previous studies that identified the presence of cognitive dysfunction in Asian patients with MDD. Overall, the mean total PDQ-D score in our study was 22.6 ± 16.2, which fell within the 3^rd^ quartile of severity. PDQ-D was also reported in the PERFORM-K study, with a mean value of 29.9 ± 18.6, also falling within the 3^rd^ quartile of severity [15]. The higher mean PDQ-D score in PERFORM-K is likely due to the higher depression severity in the PERFORM-K study population compared to the present study.

Similar to PERFORM-K, we report higher levels of perceived cognitive dysfunction in the domains of “attention/concentration” and “planning/organisation” than “prospective memory” and “retrospective memory”. While objective performance in neuropsychological tests and subjectively-reported cognitive dysfunction have been shown to have little correlation [24], the perceived cognitive dysfunction described by patients with MDD in our study using a patient-reported tool provides complementary evidence to previous studies that reported cognitive dysfunction associated with MDD using neuropsychological tests [25]. Overall, these findings suggest that cognitive dysfunction is present in MDD in both the acute and remission phases, with more severe impairment in some cognitive domains than others.

Furthermore, our study showed that younger patients (21-40 years of age) had worse perceived cognitive dysfunction when compared to patients between the ages of 41 to 60. It is possible that younger people may be more aware of cognitive deficits, whereas older people would associate them more easily with age. Cognitive dysfunction can have a substantial impact on work performance and productivity in patients with MDD [26], particularly for those in their productive years and likely to be working [27]. Physicians should therefore evaluate and monitor cognitive dysfunction especially in young, employed patients with MDD.

In addition to depression severity and cognitive dysfunction, functional disability was evaluated in all three Asian studies, including the present study [15, 23]. Similarly to perceived cognitive dysfunction, functional disability scores were lower in the present study than in SAAD or PERFORM-K (mean total SDS scores were 11.3 ± 7.9 in the present study, 17.1 ± 8.0 in SAAD and 16.7 ± 8.6 in PERFORM-K) [15, 23]. This may again be related to the differences in the populations considered by the three studies: SAAD and PERFORM-K represent a more severe MDD population, whereas this study reflects a population with less severe depression, the majority of whom were being treated prior the study visit and may therefore already be recovering from their depressive episode.

The majority of patients in our study, who were on various stages of MDD treatment, experienced ‘somewhat’ to ‘extreme’ difficulty with working, taking care of things at home or getting along with people. Thus, it is clear that patients with MDD on treatment can experience functional disability affecting work, family life and leisure activities. In addition, these patients reported clinically relevant perceived cognitive dysfunction as assessed by a mean total PDQ-D cut-off score of ≥20, which hints at the role cognitive dysfunction may play a role in functional disability and the limitations of most available treatments to address these.

A trend observed in the two previous Asian studies and the current study was that patients reported worse functional disability in “work/school”, than in “social life/leisure” or “family/home life” [15, 23]. This contrasts with Western reports of worse functional disability in the “social life/leisure” domain when compared to other domains [28], which could suggest differences in the emphasis placed on work or social functioning in Asian and Western cultures, or reflect differences in the perception of functional disability between Asian and Western patients.

In addition to characterising depression severity, perceived cognitive dysfunction and functional disability, the three studies also investigated the relationships between depression severity, perceived cognitive dysfunction and functional disability.

Based on these, a clear correlate of perceived cognitive dysfunction across all three Asian studies, including the present study, is depression severity. Studies that evaluated the association between depression severity and cognitive dysfunction using objective neuropsychological tests have identified significant correlations between depression severity and cognitive functioning in the specific domains of episodic memory, executive function, and processing speed [11]. Results of the Asian studies that evaluated perceived cognitive dysfunction using patient-reported outcomes provide complementary evidence for the relationship between depression severity and cognitive dysfunction. The SAAD showed that depression severity was independently associated with both subjective concentration deficits (OR: 2.72, 95% CI 1.68-4.39) and subjective memory deficits (OR: 2.42, 95% CI 1.62-3.64) [13]. Depression severity was similarly correlated with perceived cognitive dysfunction in both the PERFORM-K study (Pearson’s correlation coefficient: 0.41) [15] and in the present study (Pearson’s correlation coefficient: 0.69). Since affective symptoms, such as depression or anxiety, at the time of cognitive assessment can influence patients’ subjective reporting of their cognitive symptoms [24, 29], the different impact of depression severity on actual cognitive performance or perceived cognitive dysfunction is worth exploring.

Depression severity has also shown to correlate with functional disability. A cross-cultural study observed that patients with worse depression severity assessed by the PHQ-9 reported greater functional disability across the domains of mental health, social functioning and general health perception assessed by the Short-Form 20-item survey [30]. Similar trends have been observed in Asian studies: in PERFORM-K, patients with worse depression severity reported greater functional disability and work impairment [15]. Another subgroup analysis of SAAD showed that both physician-assessed depression severity and patient-reported depression severity were associated with functional disability (*p*<0.01 for both associations) [31]. The present study again confirmed these associations, and reported a high correlation between patient-reported depression severity and functional disability (Pearson’s correlation coefficient: 0.72).

Given the correlations between the three variables, our study further explored the multi-directional relationship through the use of ANCOVA models. The ANCOVA results showed that after controlling for depression severity, one of the factors significantly associated with functional disability was perceived cognitive dysfunction (*p*<0.001). Our results were consistent with a large prospective observational cohort study conducted across 5 countries in Europe that similarly reported an association between worse patient-reported depression severity and overall functional impairment (*p*<0.001) [32]. These studies suggest that over and above the impact of depression severity, perceived cognitive dysfunction also is a key determinant of functionality disability. Indeed, existing literature show that improvements in depressive symptoms are not necessarily followed by improvements in functional disability; functional disability has been found to be persistent, even in patients in remission [3]. It has thus been posited that different factors besides depression severity may be associated with enduring functional disability [3], including cognitive dysfunction. Functional outcomes in patients with MDD may rely on both the alleviation of depressive symptoms and remediation of cognitive impairment [25]. The results from our study, which showed an independent association between perceived cognitive dysfunction and functional disability in an MDD population with relatively low depression severity, underline the need to assess cognitive dysfunction in patients and to consider the long-term implications of continued cognitive dysfunction on patients’ recovery.

The ANCOVA results showed significant differences in functional disability between patients who were in a recurrent depressive episode and patients in a first depressive episode (*p*<0.05). Our study found that the mean total PDQ-D scores for the patient subgroups “3 previous depressive episodes” and “>3 previous depressive episodes” were both ≥20 (indicating clinically relevant perceived cognitive dysfunction). Several studies have previously reported worse cognitive dysfunction in patients with repeated depressive episodes when compared to patients in a first or second episode [33, 34]. One study in particular explored whether memory impairment is a “state” marker, reflecting the direct impact of mood on the patient’s current cognitive state, or a “trait” marker, reflecting the enduring effects of MDD on brain function [35]. This study showed that progressive memory decline occurred with increasing previous depressive episodes, with memory performance estimated to be impaired by 2-3% for each previous depressive episode, up to four episodes [35]. Therefore, past depressive episodes can influence the severity of cognitive function in future depressive episodes. The question of whether cognitive dysfunction could in fact be a mediator of worse functional disability in recurrent depressive episodes is raised and warrants further research.

Significant differences in functional disability were also reported between countries (*p*<0.05). Interpretation of these differences is however limited by the observational nature of the present study.

The lack of awareness amongst physicians and patients of cognitive dysfunction as a core feature of MDD can result in under-reporting of cognition-related symptoms by patients [18], particularly among older patients with poor reporting abilities [36], as well as suboptimal evaluation by physicians [37]. The present study showed that use of a patient-reported outcome measure can provide valuable information about the patient’s cognitive functioning, which is an important determinant of functional outcomes in patients with MDD. The patient self-report tool (PDQ-D) is easy to administer in the clinical setting, and can provide information on the experience and impact of cognitive dysfunction on patients’ everyday lives that are complementary to information gained from objective neuropsychological tools. In addition, a validation study of the Korean version of the PDQ-D showed that sub-scores of the ‘Attention/Concentration’, ‘Retrospective Memory’ and ‘Prospective Memory’ domains correlated with patients’ functional disability, sick leave days and quality of life (as measured by the European Quality of Life 5-Dimension questionnaire), reflecting the predictive value of the PDQ-D on patients’ daily functioning, work productivity and overall quality of life [38]. Given the persistence of cognitive dysfunction even in clinical remission of patients, and since patients’ experience of these symptoms is key in their functional recovery [18], use of a patient-reported tool in clinical settings should be considered in the holistic management of patients with MDD that aims to achieve not only the remission of mood symptoms but also functional recovery.

This study had several limitations. Firstly, conclusive interpretation of causality between perceived cognitive dysfunction, depression severity and functional disability was not possible due to the cross-sectional nature of the study design with patients assessed during a single study visit. Secondly, the present study was conducted across 6 Asian countries, thus results of this publication were not necessarily generalisable to any specific country. Furthermore, the majority of the present study population reported mild-to-moderate depression, thus results were also not necessarily generalisable to patients with severe MDD. Thirdly, since depression itself may influence the perception of cognitive function or functional status [8], future studies using objective measures of cognitive dysfunction and functional disability are required to validate results from the present study. Finally, other aspects of cognitive dysfunction and functional outcomes, such as social cognition and the impact of MDD on specific domains of functional disability in Asian patients, are important aspects of MDD to explore.

## CONCLUSION

This study highlights the presence of cognitive dysfunction amongst Asian patients with MDD, and describes the profile of Asian patients with different degrees of perceived cognitive dysfunction. The results of our study contribute to current knowledge of how cognitive dysfunction relates to depression severity and patient functioning. Physicians should consider the evaluation of cognitive dysfunction as an integral part of MDD management, particularly in light of treatment goals encompassing functional recovery beyond remission of clinical symptoms.

## Figures and Tables

**Table 1:** Patient demographics and medical history.

	***n***	**Total**
**Age, Mean ± S.D.**	664	46.5 ± 12.5
**Gender, n (%)**	664	
**Male**		224 (33.7%)
**Female**		440 (66.3%)
**Country, n (%)**	664	
**Indonesia**		21 (3.2%)
**Malaysia**		211 (31.8%)
**Philippines**		2 (0.3%)
**Singapore**		36 (5.4%)
**Taiwan**		226 (34.0%)
**Thailand**		168 (25.3%)
**Clinic Setting, n (%)**	664	
**Outpatient**		638 (96.1%)
**Inpatient**		26 (3.9%)
**Marital Status, n (%)**	664	
**Single**		171 (25.8%)
**Married or living as a couple**		391 (58.9%)
**Divorced/separated**		75 (11.3%)
**Widowed**		27 (4.1%)
**Living Area, n (%)**	664	
**City**		540 (81.3%)
**Small town**		103 (15.5%)
**Rural**		21 (3.2%)
**Total Number of School, College or University Education, Mean ± S.D.**	664	12.8 ± 4.7
**Main work status, n (%)**	664	
**Working^a^, students or homemakers**		562 (84.6%)
**Non-working**		58 (8.7%)
**Retired**		37 (5.6%)
**Other**		7 (1.1%)
**MDD History**		
**Time since beginning of depressive episode, n (%)**	664	
**<1 week**		20 (3.0%)
**1-2 weeks**		18 (2.7%)
**2-4 weeks**		42 (6.3%)
**4-8 weeks**		38 (5.7%)
**>8 weeks**		546 (82.2%)
**Current episode is patient’s first depressive episode, n (%)**	664	342 (51.5%)
		
**Number of previous depressive episodes, n (%)**	322^b^	
**1 depressive episode**		113 (35.1%)
**2 depressive episodes**		94 (29.2%)
**3 depressive episodes**		58 (18.0%)
**>3 depressive episodes**		57 (17.7%)
**Last depressive episode occurred within the last 12 months, n (%)**	322^b^	95 (29.5%)
**Last episode was treatment with an antidepressant, n (%)**	322^b^	294 (91.3%)
**Patient achieved clinical remission after last episode, n (%)**	322^b^	264 (82.0%)
**MDD Treatment**		
**Current depressive episode being treated before this visit^c^**	664	576 (86.7%)
**Number of treatments for current depressive episode before this visit^d^**	576	
**On 1 treatment only**		451 (78.3%)
**On 2 treatments**		114 (19.8%)
**On 3 treatments**		11 (1.9%)
**Patients were initiated, switched or maintained treatment, n (%)**	664	
**Initiated**		88 (13.3%)
**Switched**		79 (11.9%)
**Maintained**		497 (74.8%)
**Medical History**		
**Patients with clinically significant symptoms of anxiety in the current episode, n (%)**	664	371 (55.9%)
**Patients with anxiety disorders**	664	141 (21.2%)
**Patients with chronic medical condition**	664	354 (53.3%)
**Patients with functional syndromes**	664	324 (48.8%)
**Sick leave prescribed related to MDD**	52^e^	49 (94.2%)

^a^ “Working” patients were patients who had paid work, non-paid work (e.g. charity work or volunteer work), or were self-employed

^b^ Patients with a previous depressive episode

^c^ This includes patients who discontinued in past 2 weeks prior current study visit

^d^ Patients whose current depressive episode was being treated before this visit

^e^ Patients who were prescribed sick leave

**Table 2:** Depression severity, perceived cognitive dysfunction and functional impairment.

	*n*	Values
**CGI-S**	664	
Total Score (mean ± SD)		3.28 ± 0.99
By category:		
Normal (1)		26 (3.9%)
Mild (2-3)		362 (54.5%)
Moderate (4)		207 (31.2%)
Severe (5-7)		69 (10.4%)
**PHQ-9**	664	
Total Score (mean ± SD)		11.27 ± 6.92
By category:^a^		
No depression (0-4)		124 (18.7%)
Mild depression (5-9)		179 (27.0%)
Moderate depression (10-14)		143 (21.5%)
Moderately severe depression (15-19)		121 (18.2%)
Severe depression (20-27)		97 (14.6%)
**PDQ-D**	664	
Total Score (mean ± SD)		22.59 ± 16.16
By category:		
1^st^ quartile (0-9)		4.32 ± 2.99
2^nd^ quartile (10-19)		14.39 ± 2.89
3^rd^ quartile (20-31)		25.21 ± 3.66
4^th^ quartile (32-80)		44.37 ± 10.58
By domain:		
Attention/Concentration		6.21 ± 4.64
Retrospective Memory		5.39 ± 4.41
Prospective Memory		4.99 ± 3.88
Planning/Organisation		6.00 ± 4.93
**SDS**	598^b^	
Total Score (mean ± SD)		11.31 ± 7.94
By domain:		
Work/School Work	526^c^	4.21 ± 3.03
Social life/Leisure Activities	664	3.86 ± 2.93
Family Life/Home Responsibilities	664	3.83 ± 2.95

^a^PHQ-9 categories of severity were defined *a priori*: 0 to 4 (“no depression”), 5 to 9 (“mild depression”), 10 to 14 (“moderate depression”), 15 to 19 (“moderately severe depression”), 20 to 27 (“severe depression”).

^b^Excluding patients who had not worked/studied in the past week (n=66)

^c^Excluding patients who had not answered (n=72) and patients who had not worked/studied in the past week (n=66)

**Table 3:** Summary of ANOVA (*p*-values) to identify factors associated with functional disability (SDS).

**-**	**SDS Total**	**SDS Work**	**SDS Social**	**SDS Family**
**Country**	0.000**	0.000**	0.000**	0.000**
**Gender**	0.079	0.133	0.039*	0.931
**Age**	0.000**	0.000**	0.000**	0.000**
**Marital Status**	0.002*	0.002*	0.008*	0.455
**Education**	0.008*	0.083	0.008*	0.140
**Recurrent Depressive Episode**	0.094	0.155	0.021*	0.028*
**Hospitalisation**	0.011*	0.023*	0.016*	0.022*
**Sick Leave**	0.000**	0.000**	0.000**	0.000**
**CGI-S Total Score**	0.000**	0.000**	0.000**	0.000**
**PHQ-9 Total Score**	0.000**	0.000**	0.000**	0.000**
**At Least 1 Chronic Medical Condition**	0.068	0.769	0.029*	0.574
**At Least 1 Functional Syndrome**	0.000**	0.012*	0.000**	0.000**
**At Least 1 Anxiety Disorder**	0.992	0.996	0.696	0.965
**At Least 1 Concomitant Mental Disorder**	0.389	0.500	0.712	0.242
**Antidepressant Treatment**	0.000**	0.000**	0.000**	0.000**
**PDQ-D Total Score**	0.000**	0.000**	0.000**	0.000**
**Treatment Maintained**	0.000**	0.002*	0.000**	0.000**
**Treatment Started**	0.000**	0.012*	0.000**	0.000**
**Treatment Switched**	0.111	0.074	0.151	0.074

**p<0.001; *p<0.05

**Table 4:** Summary of ANCOVA models (*p*-values) to identify factors associated with functional disability (total SDS score).

**-**	**ANCOVA Model 1^§^**	**ANCOVA Model 2^§^**	**ANCOVA Model 3^§^**
**PDQ-D Total Score**	0.000**	n/a	0.000**
**Country**	0.003*	0.000**	0.001*
**Recurrent Depressive Episode**	0.013*	0.010*	0.021*
**CGI-S Total Score**	0.056	n/a	n/a
**Gender**	0.109	0.066	0.068
**Age**	0.126	0.087	0.163
**Education**	0.160	0.222	0.132
**Sick Leave**	0.161	0.122	0.163
**Antidepressant Treatment**	0.212	0.283	0.167
**Marital Status**	0.829	0.760	0.815

^§^ANCOVA model 1 was built using factors associated with functional disability (total SDS score) in univariate analysis (*p*<0.05 in ANOVA). ANCOVA model 2 was then built without both PDQ-D and CGI-S. Finally, ANCOVA model 3 was built with PDQ-D, but without CGI-S. Summary of results are presented as p-values. PHQ-9 was an adjustment covariate in all 3 ANCOVA models. **p<0.001; *p<0.05
